# Response of soil microbial community structure and function to different altitudes in arid valley in Panzhihua, China

**DOI:** 10.1186/s12866-022-02500-6

**Published:** 2022-04-02

**Authors:** Runji Zhang, Xianrui Tian, Quanju Xiang, Petri Penttinen, Yunfu Gu

**Affiliations:** Department of Microbiology, College of Resources, Sichuan Agricultural University, Chengdu, 611130 China

**Keywords:** Arid valley, Altitudinal gradients, Soil properties, Microbial communities, Functional prediction

## Abstract

**Background:**

Altitude affects biodiversity and physic-chemical properties of soil, providing natural sites for studying species distribution and the response of biota to environmental changes. We sampled soil at three altitudes in an arid valley, determined the physic-chemical characteristics and microbial community composition in the soils, identified differentially abundant taxa and the relationships between community composition and environmental factors.

**Results:**

The low, medium and high altitudes were roughly separated based on the physic-chemical characteristics and clearly separated based on the microbial community composition. The differences in community composition were associated with differences in soil pH, temperature, and SOC, moisture, TN, TP, AN, AP and SMBC contents. The contents of organic and microbial biomass C, total and available N and available P, and the richness and diversity of the microbial communities were lowest in the medium altitude. The relative abundances of phyla Proteobacteria, Gemmatimonadetes, Actinobacteria and Acidobacteria were high at all altitudes. The differentially abundant amplified sequence variants (ASVs) were mostly assigned to Proteobacteria and Acidobacteria. The highest number of ASVs characterizing altitude were detected in the high altitude. However, the predicted functions of the communities were overlapping, suggesting that the contribution of the communities to soil processes changed relatively little along the altitude gradient.

**Conclusions:**

The low, medium and high altitudes were roughly separated based on the physicochemical characteristics and clearly separated based on the microbial community composition. The differences in community composition were associated with differences in soil pH, temperature, and SOC, moisture, TN, TP, AN, AP and SMBC contents.

**Supplementary Information:**

The online version contains supplementary material available at 10.1186/s12866-022-02500-6.

## Background

Altitude affects biodiversity and physic-chemical properties of soil; the plant cover, soil properties and climate change dramatically with altitude in mountain ecosystems, providing natural sites for studying species distribution and the response of biota to environmental changes [1]. Knowledge on altitude related biodiversity patterns is important for understanding the impacts of climate change on ecosystems [2].

Microbial communities play an important role in C and nutrient cycles, respond rapidly to and are affected by environmental changes [3, 4]. The activity and structure of microbial communities are affected by soil type, temperature, vegetation and other abiotic and biological factors [5]. To date, the research on microbial communities in mountain ecosystems has mostly focused on plateaus and frozen soils [6, 7]. The results on microbial diversity along altitude gradients have been non-uniform: diversity has been found to decrease with altitude, to be highest at mid-altitude, and to show no clear patterns of change [1, 8–10]. At lower elevations, soil microbes have shown clear altitudinal distribution patterns [11, 12]. In Changbai Mountain in China, a small difference in elevation indirectly controlled the composition of soil microbial community, and the relationship between altitude and soil microbial community was not linear [13]. The changes in community composition and microbial biomass and activity were accompanied with changes in altitude, vegetation type and soil physic-chemical properties, e.g. C and N content and pH [14–16]. Therefore, the spatial distribution patterns of soil microbial communities are associated with both present and historical factors, i.e. soil properties and altitude, respectively [13].

Panzhihua in the south of Sichuan, China, at the interchange between Jinsha River and Yalong River, has a subtropical climate with abundant rainfall and a hot rainy season. However, high evapotranspiration due to strong sunshine and valley winds result in an arid local climate in the arid valley areas [17, 18]. Soil in Panzhihua is typically acid red soil, and the area of soil with pH lower than 5.5 accounts for almost 16% of the total soil area [19]. Information on the effects of altitude and environmental factors on soil microbial communities in arid valleys is still limited. The temperature and air humidity in this region vary considerably within a small range of altitude difference. We expected that the soil microbial community composition would vary accordingly. In this study, we sampled soil at three altitudes in an arid valley, determined the physico-chemical characteristics and microbial community composition in the soils, identified differentially abundant taxa and the relationships between community composition and environmental factors, providing a basis for the understanding of soil ecosystems in arid valleys.

## Results

### Soil physic-chemical characteristics

The soil pH and temperature were lowest and AK content was highest at the high altitude (2000 m a.s.l.) (*P* < 0.05) (Table 1). Soil moisture was lowest and TK content was highest at the low altitude (1600 m a.s.l.) (*P* < 0.05). TP content increased with increasing altitude (*P* < 0.05). SOC, TN, AP, AN, and SMBC contents were highest at the high altitude and lowest at the medium altitude (1800 m a.s.l.) (*P* < 0.05). SMBN content was lowest at the medium altitude. Based on the physicochemical properties, the soil samples were roughly separated according to the altitude (Fig. S 1). All the other properties except pH, moisture and microbial biomass N content showed collinearity (|*r2*|> 0.7) (Table S 1).

**Table 1:** The soil properties at different sites along the altitudes

	Low	Medium	High
pH	5.45 ± 0.42^a^	5.40 ± 0.26^a^	5.17 ± 0.13^b^
SOC (g·kg^−1^)	10.20 ± 1.02^b^	6.30 ± 0.67^c^	16.38 ± 1.34^a^
Moisture (%)	27.25 ± 1.54^b^	32.09 ± 1.39^a^	33.31 ± 1.91^a^
TN (g·kg^−1^)	2.48 ± 0.49^b^	1.87 ± 0.15^c^	3.12 ± 0.19^a^
TP (g·kg^−1^)	1.07 ± 0.49^c^	1.16 ± 0.10^b^	1.33 ± 0.06^a^
TK (g·kg^−1^)	6.75 ± 0.20^a^	6.13 ± 0.76^b^	5.55 ± 0.48^b^
AN (mg·kg^−1^)	31.64 ± 7.43^b^	21.81 ± 3.43^c^	46.26 ± 2.53^a^
AP (mg·kg^−1^)	7.41 ± 2.73^b^	4.61 ± 2.70^c^	33.77 ± 7.50^a^
AK (mg·kg^−1^)	327.63 ± 78.82^b^	412.87 ± 257.69^b^	1094.40 ± 121.32^a^
Temperature (°C)	25.07 ± 0.54^a^	24.32 ± 1.59^a^	20.91 ± 0.99^b^
SMBC (mg·kg^−1^)	90.67 ± 13.05^b^	62.08 ± 9.68^c^	156.80 ± 4.92^a^
SMBN (mg·kg^−1^)	5.24 ± 1.26^a^	3.37 ± 0.63^b^	5.35 ± 0.61^a^

Data are average ± SEM (n = 3). Different letters in a column denote statistically significant differences (*P* < 0.05). *SOC* Soil organic carbon, *MO* Moisture, *TN* Total nitrogen, *TP* Total phosphorus, *TK* Total potassium, *AN* Available nitrogen, *AP* Available phosphorus, *AK* Available potassium, *ST* Soil temperature, *SMBC* Soil microbial biomass carbon, *SMBN* soil microbial biomass nitrogen. High, altitude 2000 m a.s.l.; medium, altitude 1800 m a.s.l.; low, altitude 1600 m a.s.l

### Microbial communities

**Fig. 1:**
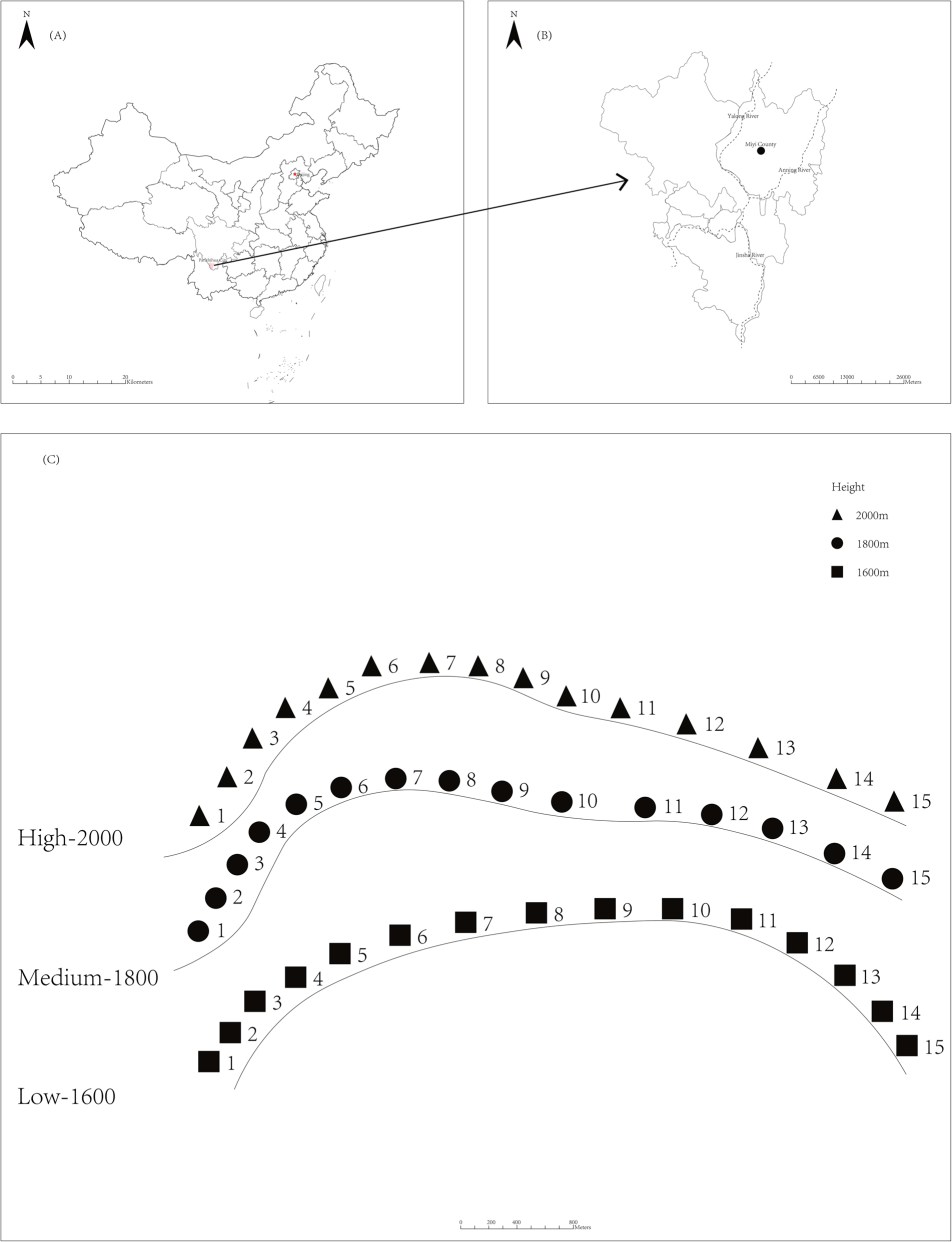
Location of the arid valley in Panzhihua city and sampling sites. High, altitude 2000 m a.s.l.; medium, altitude 1800 m a.s.l.; low, altitude 1600 m a.s.l

The richness of the microbial communities was highest at the low altitude and lowest at the medium altitude (*P* < 0.05) (Table 2). The diversity was lowest at the medium altitude (*P* < 0.05). The relative abundances of phyla Proteobacteria were above 40%, and those of Gemmatimonadetes and Actinobacteria were above 12% at all altitudes (Fig. 1A). The relative abundances of genera *Sphingomonas*, *Gemmatimonas* and *Saccharimonadales* were high at all altitudes (Fig. 1B).

**Table 2:** Alpha diversity of bacterial diversity at different sites along the altitudes

Altitude	Chao1	Shannon
High	1970.45 ± 306.98^b^	0.996 ± 0.001^a^
Medium	1645.68 ± 177.23^c^	0.992 ± 0.002^b^
Low	2121.47 ± 193.67^a^	0.997 ± 0.001^a^

Data are average ± SEM. Different letters in a column denote statistically significant differences (*P* < 0.05). High, altitude 2000 m a.s.l.; medium, altitude 1800 m a.s.l.; low, altitude 1600 m a.s.l

**Fig. 2:**
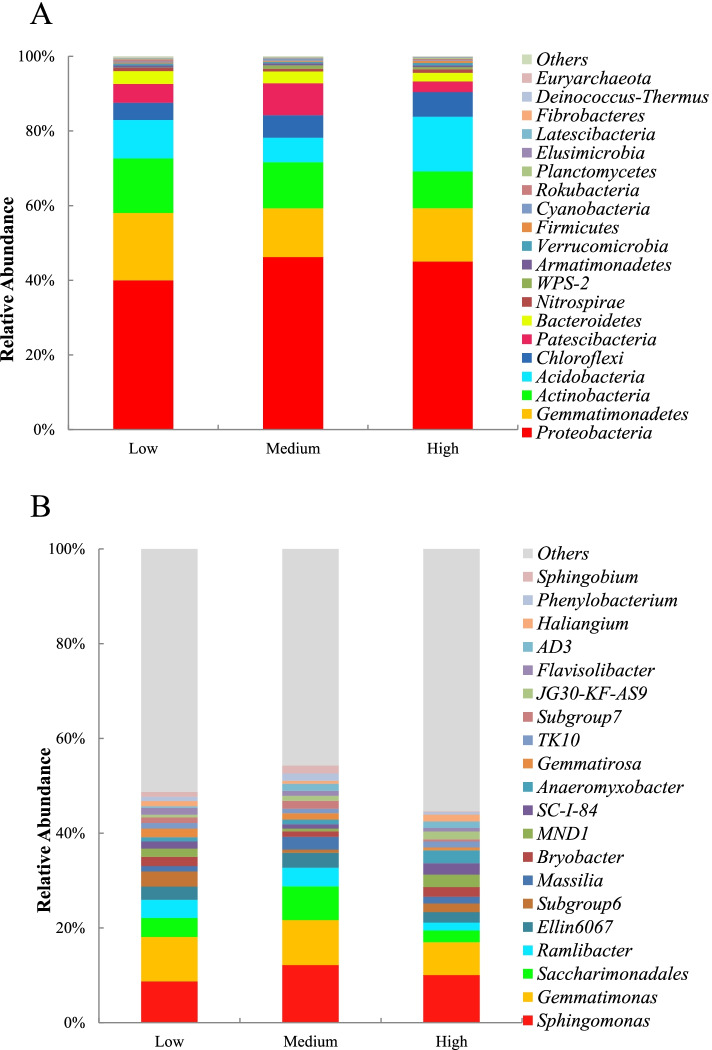
Relative abundance of dominant soil microbial community in different altitudes: **A** genus level; **B** phylum level. Low: High, altitude 2000 m a.s.l.; medium, altitude 1800 m a.s.l.; low, altitude 1600 m a.s.l

Out of the 58802 amplified sequence variants (ASVs), the 3674 ASVs with an observed mean abundance higher than one were included in the Permanova tests, distance-based redundancy analysis (DBRDA) and differential abundance analysis. Aitchison distance based Permanova and pairwise Permanova showed that the communities at different altitudes were distinct (Permanova pseudo-F = 12.7, *p* = 0.001; permutest *p* = 0.309) (Table 3). Across all the samples, the differences in community composition were associated with differences in soil pH, temperature, and SOC, moisture, TN, TP, AN, AP and SMBC contents (*P* < 0.05) (Fig. 2, Table 4). In the reduced models, the p-value of SMBN ranged from 0.001 to 0.1. At low altitude, the differences in community composition were associated with differences in pH and temperature, at medium altitude, with differences in TN content, and at high altitude, with differences in soil microbial biomass C content (Table S 2).

**Table 3:** Pairwise Permanova between bacterial community compositions at different sites along the altitudes

	F Model	R2	p adjusted
Low vs Medium	11.37	0.29	0.00
Low vs High	13.00	0.32	0.00
Medium vs High	13.89	0.33	0.00

High, altitude 2000 m a.s.l.; medium, altitude 1800 m a.s.l.; low, altitude 1600 m a.s.l

**Table 4:** The relationships between community composition and standardized soil properties across all samples from the three altitudes, tested using distance-based redundancy analysis

	F	Pr(> F)
pH	2.9–3.1	**0.005–0.001**
SOC	11.1	**0.001**
MO	7.7–8.6	**0.001**
TN	9.6	**0.001**
TP	2.2	**0.012**
TK	0.95	0.429
AN	8.3	**0.001**
AP	8.3	**0.001**
AK	1.4	0.154
Temperature	4.0	**0.001**
SMBC	1.6	0.092
SMBN	1.6–8.9	0.1–**0.001**

*SOC* Soil organic carbon, *MO* Moisture, *TN* Total nitrogen, *TP* Total phosphorus, *TK* Total potassium, *AN* Available nitrogen, *AP* Available phosphorus, *AK* Available potassium, Temperature: Soil temperature, *SMBC* Soil microbial biomass carbon, *SMBN* soil microbial biomass nitrogen

Altogether 354 differentially abundant ASVs were detected between the low and medium altitudes, 384 between the low and high altitudes, and 408 between the medium and high altitudes (Table S 2). ASVs differentially abundant in comparing one altitude to both the other altitudes were taken as characteristic to the altitude. Ten and three ASVs were enriched and depleted, respectively, in the low altitude compared to both the medium and high altitude (Table 5). The relative abundance of the ten and three ASVs characteristic to low altitude decreased and increased, respectively, constantly along the altitude. One enriched ASV was characteristic to the medium altitude (Table 5). Altogether 152 enriched and 32 ASVs were characteristic to the high altitude (Table 5). Most of the altitude characteristic ASVs were assigned to phyla Proteobacteria and Acidobacteria, and at lower taxonomic levels to uncultured and unclassified taxa (Table 5). Out of the well-defined taxa, *Anaeromyxobacter, Gemmatimonas, Sphingomonas, Bryobacter, Flavisolibacter* and *Phenylobacterium* included both enriched and depleted ASVs (Table 5).

**Table 5:** Number of ASVs characteristic to low, medium and high altitudes and their taxonomic affiliation

		**Low**		**Medium**		**High**	
**Phylum**	**Taxon**	Enriched	Depleted	Enriched	Depleted	Enriched	Depleted
Acidobacteria	*Bryobacter*					7	2
	*Candidatus* Solibacter					6	
	*Granulicella*					1	
	*Occallatibacter*					1	
	unclassified Acidobacteriales					1	
	uncultured Acidobacteriaceae (Subgroup 1)					4	
	uncultured Acidobacteriales	1				16	1
	Pyrinomonadaceae clade RB41					3	
	Subgroup 7	1				2	1
	Subgroup 6					4	
	*Angustibacter*					1	
Actinobacteria	*Terrabacter*					1	
	unclassified Kineosporiaceae					1	
	uncultured Frankiales	1					1
	Class MB					1	
	*Gaiella*					1	
	*Patulibacter*						1
	uncultured Gaiellales					3	
Bacteroidetes	*Flavisolibacter*					1	2
AD3	Phylum AD3					2	
Chloroflexi	uncultured Roseiflexaceae					1	
	Family JG30					3	
	Order B12					1	
	Order C0119					2	
	Cluster TK10					3	
Gemmatimonadetes	*Gemmatimonas*	1				7	3
	*Gemmatirosa*					3	
	unclassified Gemmatimonadaceae	2	1			4	3
	uncultured Gemmatimonadaceae					11	
Nitrospirae	*Nitrospira*					3	
Patescibacteria	family Saccharimonadales					1	1
Proteobacteria	*Bradyrhizobium*					1	
	*Phenylobacterium*	1				1	1
	*Pseudolabrys*					1	
	*Sphingobium*						2
	*Sphingomonas*					8	2
	unclassified Xanthobacteraceae					2	
	uncultured Alphaproteobacteria					1	
	uncultured Caulobacteraceae					2	
	uncultured Elsteraceae					2	
	uncultured Elsterales					5	
	uncultured Rhodospirillaceae					1	
	uncultured Xanthobacteraceae						1
	*Anaeromyxobacter*				1	9	2
	*Haliangium*					5	
	*Pajaroellobacter*						1
	family A21b					1	
	*Burkholderia*					1	
	*Caenimonas*					1	
	order Ellin6067	2				4	6
	*Lysobacter*					1	
	*Massilia*	1					1
	MND1		2			3	
	*Ramlibacter*					1	1
	Family SC					5	
	unclassified Methylophilaceae					1	
Verrucomicrobia	*Candidatus* Udaeobacter					1	

## Discussion

The diversity and activity of microbial communities depend on temperature and other climatic variables that change substantially along altitude [1, 2]. Thus, altitude gradients provide natural sites to assess the effects of environmental change on the communities. To date, the diversity, composition and function of microbial communities along altitude gradients under various climates have received attention [5, 20, 21]. To estimate whether the communities in arid climate vary similarly, we studied microbial community composition and the relationships between community composition and environmental factors along an altitude gradient in an arid valley in Panzhihua, China.

With the increase of altitude, the change in climate and vegetation result in changes in soil physico-chemical properties [14, 22, 23]. Below tree line, the SOC and TN contents increased with the increase of altitude [14, 24]. In our study, the low and high-altitude sites in the arid valley were clearly different based on the physical properties. The available K and available P contents were over two to over seven times higher in the high than in the lower altitudes. Out of the measured chemical properties, the contents of organic and microbial biomass C, total and available N and available P were different at different altitudes; however, the contents were lowest in the medium altitude and highest in the high altitude. Similarly, the richness and diversity of the microbial communities were lowest in the medium altitude. Accumulation of SOC at the high-altitude site was possibly due to the lower temperature that is known to slow down soil respiration [25]. The differences in SMBC content implied that the absolute abundance of the microbial community members were highest at the high altitude. The availability of N and P decrease below pH 6 and 4.5, respectively, thus the differences in pH between sites were not expected to affect the availability of P and have only a minor effect on the availability of N. Possibly, the availability of N and P were governed by differences in plant uptake of the nutrients and in N and P cycle related microorganisms [26–28]. As the total P content increased with the increasing altitude, the differences in the P contents may have been due to differences in bedrock along the altitude gradient [29, 30].

The relative abundances of phyla Proteobacteria, Gemmatimonadetes, Actinobacteria and Acidobacteria, all among the OTU richest phyla on earth [31], were high at all altitudes. Similar to earlier studies [1, 32], the microbial community compositions at the altitudes were distinct. The differentially abundant ASVs were mostly assigned to Proteobacteria and Acidobacteria. The highest number of ASVs characterizing altitude were detected in the high altitude, suggesting a bigger difference between the high and lower altitudes than that revealed by community composition only.

In assessing the relationships between the microbial community composition and environmental factors, the differences in community composition were associated with differences in soil pH, temperature, and SOC, moisture, N, P and SMBC contents. On a continental scale over a wide pH range, soil pH was the master variable in explaining variation in microbial communities [33]. Presumably, the associations of community composition with temperature, moisture, SOC and SMBC content were interlinked: lower temperature is connected with lower evaporation and lower rate of soil respiration [25], thus providing more organic C as an energy source for the soil microbial communities. The within altitude associations between the community composition and soil properties were rare, implying relatively homogenous communities and environments at the three altitudes.

The reason for this is that altitude is not a single factor that causes microbial community changes, but the comprehensive influence of soil properties should be considered. However, how to determine the specific relationship between the changes of microbial community at different altitudes and soil physical and chemical factors still needs further research.

## Conclusion

The low, medium and high altitudes were roughly separated based on the physicochemical characteristics and clearly separated based on the microbial community composition. The differences in community composition were associated with differences in soil pH, temperature, and SOC, moisture, N, P and SMBC contents.

## Methods

### Study area and soil sampling

Anning valley in Miyi, Panzhihua, Sichuan, China (E102°17′, N26º76′, 1587–2108 m a.s.l) has a typical subtropical valley climate. The mean annual temperature ranges from 15 to 29 °C, with maximum and minimum temperatures of 34 °C in May and 13 °C in January, respectively. Annual precipitation is approximately 800 to 1200 mm.

**Fig. 3:**
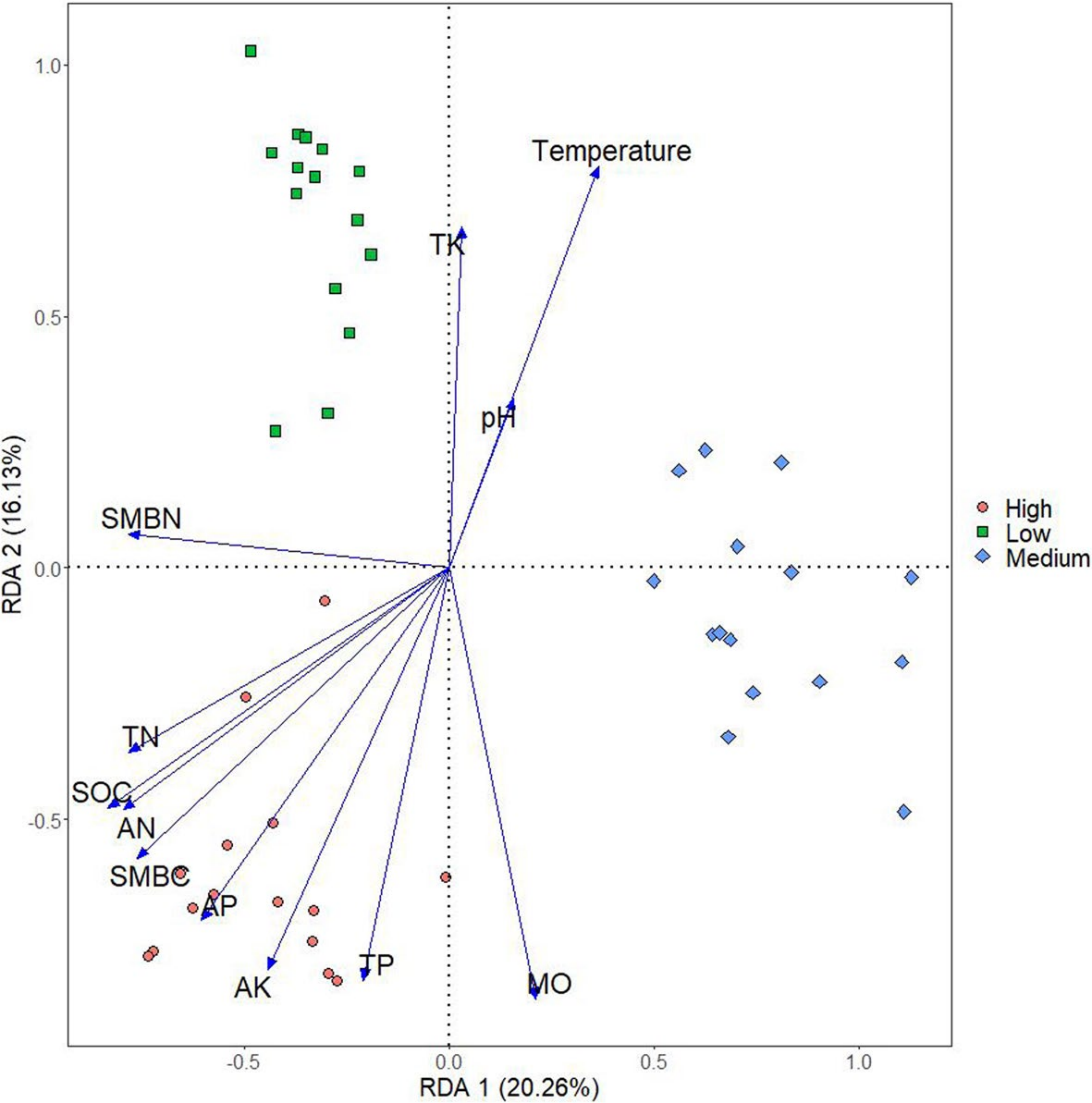
Distance-based redundancy analysis (dbRDA) of the relationships between community composition and soil properties. High, altitude 2000 m a.s.l.; medium, altitude 1800 m a.s.l.; low, altitude 1600 m a.s.l. SOC: Soil organic carbon, MO: Moisture, TN: Total nitrogen, TP: Total phosphorus, TK: Total potassium, AN: Available nitrogen, AP: Available phosphorus, AK: Available potassium, Temperature: Soil temperature, SMBC: Soil microbial biomass carbon, SMBN: soil microbial biomass nitrogen

Fifteen 20 × 20 m sampling plots with at least 100 m distance between the plots were established in July 28, 2018, at 1600, 1800 and 2000 m a.s.l (Fig. 3). Within each plot, five topsoil (0 – 10 cm) subsamples were taken from the center and each corner using a 5-cm diameter soil corer after removing litter from the soil surface by hand. The subsamples were combined into one composite soil sample per plot [34]. The composite samples were divided into two portions: the portion for physic-chemical analyses was stored at 4 °C and the portion for DNA extraction was stored at -80 °C.

### Soil physico-chemical properties

The soil samples were air-dried, ground and sieved through a 2-mm mesh. Soil pH was measured in a soil-to-water ratio of 1:1 using a glass electrode pH meter (FiveGo, Mettler Toledo, Greifensee, Switzerland). Soil organic carbon (SOC) and total nitrogen (TN) contents were determined using dichromate oxidization and Kjeldahl digestion [35], and available nitrogen (AN) as described earlier [36]. Total phosphorus (TP) and total potassium (TK) contents were determined after digestion in HF-HClO_4_. Soil moisture (MO) content was measured by drying the fresh soil samples at 105 °C until a constant weight [37]. Available phosphorus (AP) and available potassium (AK) contents were determined using sodium bicarbonate and ammonium acetate extraction [14, 38]. Soil microbial biomass C (SMBC) and biomass N (SMBN) contents were estimated by using chloroform fumigation extraction as described earlier [39].

### DNA extraction, amplification and sequencing

DNA was extracted from 1.21–1.45 g fresh weight soil (corresponding to approximately 0.50 g dry weight) using the Fast DNA Spin Kit for Soil (MP Bio medicals, Solon, OH, USA) following the manufacturer’s instructions. Concentration and quality of the extracted DNA were examined using Nano-200 spectrophotometer (Aosheng, Hangzhou, China) and agars gel electrophoresis. DNA extracts were stored at − 20 °C until further processing. The V4 hyper variable region of 16S rRNA gene was amplified using primers 515F (5′-GTGCC-AGCMGCCGCGGTAA-3′) and 806R (5′-GGACTACVSGGGTATCTAAT-3′) with adapter and barcode sequences [40]. Amplification was done in a 50 μL reaction mixture with 3 U of TaKaRa Ex Taq HS (TaKARA Shuzo Co., Shiga, Japan), 5 mM dNTP mixture (TaKARA Shuzo Co., Shiga, Japan), 2.0 mM MgCl_2_, 5 μL of 10 × Ex Taq Buffer (TaKARA Shuzo Co., Shiga, Japan), 0.6 mM of each primer, and 4.0 ng of DNA. The amplification program in an S1000 thermo cycler (Bio-Rad Laboratories, CA, USA) included an initial denaturation at 94 °C for 4 min, and 30 cycles of 15 s at 94 °C, 15 s at 55 °C and 30 s at 72 °C, and a final extension at 72 °C for 10 min.

The PCR products from three replicate amplifications per sample were pooled and purified with AxyPrep DNA Purification Kit (Axygen Biotech, Hangzhou, China), and quantified using PR omega QuantiFluor (Invitrogen, Carlsbad, CA, USA). Purified amplicons were pooled in equimolar concentrations and sequenced using MiSeq Reagent Kit V3 on an Illumina MiSeq platform (Illumina Biotech, California, USA) at Shanghai Personal Biotechnology Co. Ltd, Shanghai, China. The sequence data were submitted to NCBI Sequence Read Archive (https://www.ncbi.nlm.nih.gov/Traces/study/?acc=PRJNA663774) with accession number PRJNA663774 and login account zhangrunji.

### Bioinformatics analysis

The sequence data were processed and assigned into amplicon sequence variants (ASVs) using R package DADA2 [41]. The ASVs were assigned to taxa using Silva release 132 database (http://www.arb-silva.de). Chao1 and Shannon alpha diversity indices were calculated with QIIME software (Version 1.7.0).

### Statistical analysis

Differences were regarded as statistically significant at *P* < 0.05. Relative abundances at phylum and genus levels were visualized as percentages using the multtest package in R package vegan version 2.4.4 in R v.3.3.2. [42]. Differences in the soil properties and microbial alpha diversities were tested with two-way ANOVA using the Statistical Package for the Social Sciences (SPSS Version 19.0, SPSS Inc., Chicago, IL, USA). The associations between microbial alpha diversity and environmental factors were analyzed using Pearson correlation [43]. The heterogeneity of the variance was tested, and the original data were normalized using log-transformation or standardization prior to analysis when necessary.

For further analyses, ASVs with mean relative abundance < 1 were removed from the data. For permutational multivariate analysis of variance (Permanova) and distance-based redundancy analysis (dbRDA), zeros in the ASV relative abundance data were replaced using the count zero multiplicative (czm) method and the data was converted to proportions using R package zCompositions v1.3.4 in R v4.1.0 [44, 45]. The data were transformed to their centered logratios using R package easyCODA v0.34.3 [46]. For Permanova, a distance matrix was calculated using R package robCompositions v2.3.1 [47, 48]. Differences in community composition were tested using Permanova and pairwise Permanova with 999 permutations in R packages vegan v2.5.7 and pairwiseAdonis v.0.4, respectively [49, 50]. Homogeneity of multivariate dispersions was tested using R package vegan v2.5.7. The relationships between community composition and standardized soil properties were tested using DBRDA in R package vegan and visualized using R packages ggplot2 v3.3.5 and ggrepel v0.9.1 [51, 52]. Soil properties with |*r2*|> 0.7 were taken as collinear [53]. The properties were tested using reduced models from which the properties collinear with the target property had been excluded. Differentially abundant ASVs were determined in pairwise comparisons using Aldex2 v1.24.0 [54]. ASVs with an expected value of the Benjamini–Hochberg corrected t-test *P* < 0.05/3 (the number of comparisons) were taken as differentially abundant.

## Supplementary Information

**Additional file 1:** **Fig. S1.** Principal component analysis (PCA) of the soil sample under the treatments of different elevations. High, altitude 2000 m a.s.l.; medium, altitude 1800 m a.s.l.; low, altitude 1600 m a.s.l.

**Additional file 2:** **Table S1.** Correlation between soil properties across all samples in the three altitudes.

**Additional file 3:** **Table S2.** The relationships between community composition and standardized soil properties at low (1600 m a.s.l.), medium (1800 m a.s.l.) and high (2000 m a.s.l.) altitudes, tested using distance-based redundancy analysis.

## Data Availability

The datasets generated and/or analysed during the current study are available in the NCBI repository (https://www.ncbi.nlm.nih.gov/Traces/study/?acc=PRJNA663774) with accession number PRJNA663774 and login account zhangrunji.
