# Supplementary material for: Response of soil microbial community structure and function to different altitudes in arid valley in Panzhihua, China
Source: BMC Microbiol. 2022 Apr 2;22:86. doi: 10.1186/s12866-022-02500-6 (PMC8976301; doi:10.1186/s12866-022-02500-6)
Supplement: Supplementary file 1 — Additional file 1: Fig. S1. Principal component analysis (PCA) of the soil sample under the treatments of different elevations. High, altitude 2000 m a.s.l.; medium, altitude 1800 m a.s.l.; low, altitude 1600 m a.s.l. [file 12866_2022_2500_MOESM1_ESM.docx]

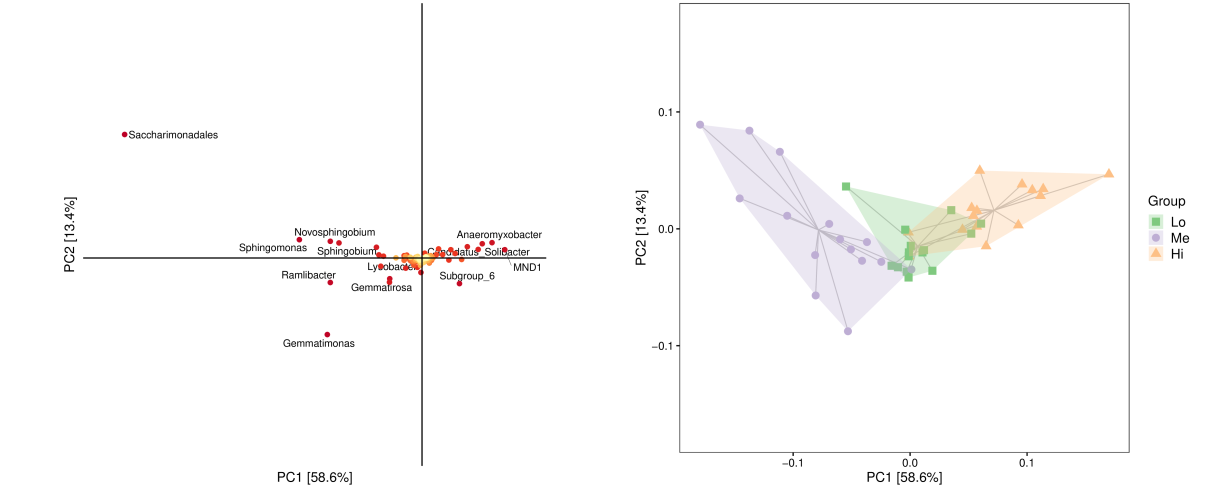


Fig. S1 Principal component analysis (PCA) of the soil sample under the treatments of different elevations. High, altitude 2000 m a.s.l.; medium, altitude 1800 m a.s.l.; low, altitude 1600 m a.s.l.
