# Supplementary material for: Response of soil microbial community structure and function to different altitudes in arid valley in Panzhihua, China
Source: BMC Microbiol. 2022 Apr 2;22:86. doi: 10.1186/s12866-022-02500-6 (PMC8976301; doi:10.1186/s12866-022-02500-6)
Supplement: Supplementary file 2 — Additional file 2: Table S1. Correlation between soil properties across all samples in the three altitudes. [file 12866_2022_2500_MOESM2_ESM.docx]

Table S1. Correlation between soil properties across all samples in the three altitudes.

|  | pH | SOC | MO | TN | TP | TK | AN | AP | AK | Temperature | SMBC | SMBN |
| --- | --- | --- | --- | --- | --- | --- | --- | --- | --- | --- | --- | --- |
| pH | 1.00 | -0.34 | -0.18 | -0.20 | -0.43 | 0.37 | -0.41 | -0.33 | -0.31 | 0.38 | -0.25 | 0.20 |
| SOC | -0.34 | 1.00 | 0.24 | 0.83 | 0.60 | -0.42 | 0.88 | 0.89 | 0.77 | -0.68 | 0.93 | 0.55 |
| MO | -0.18 | 0.24 | 1.00 | 0.21 | 0.59 | -0.56 | 0.21 | 0.42 | 0.52 | -0.56 | 0.38 | -0.09 |
| TN | -0.20 | 0.83 | 0.21 | 1.00 | 0.44 | -0.26 | 0.82 | 0.74 | 0.67 | -0.61 | 0.84 | 0.60 |
| TP | -0.43 | 0.60 | 0.59 | 0.44 | 1.00 | -0.79 | 0.60 | 0.73 | 0.69 | -0.66 | 0.59 | -0.06 |
| TK | 0.37 | -0.42 | -0.56 | -0.26 | -0.79 | 1.00 | -0.38 | -0.57 | -0.52 | 0.63 | -0.44 | 0.12 |
| AN | -0.41 | 0.88 | 0.21 | 0.82 | 0.60 | -0.38 | 1.00 | 0.80 | 0.67 | -0.63 | 0.85 | 0.44 |
| AP | -0.33 | 0.89 | 0.42 | 0.74 | 0.73 | -0.57 | 0.80 | 1.00 | 0.86 | -0.79 | 0.89 | 0.40 |
| AK | -0.31 | 0.77 | 0.52 | 0.67 | 0.69 | -0.52 | 0.67 | 0.86 | 1.00 | -0.86 | 0.79 | 0.32 |
| Temperature | 0.38 | -0.68 | -0.56 | -0.61 | -0.66 | 0.63 | -0.63 | -0.79 | -0.86 | 1.00 | -0.78 | -0.32 |
| SMBC | -0.25 | 0.93 | 0.38 | 0.84 | 0.59 | -0.44 | 0.85 | 0.89 | 0.79 | -0.78 | 1.00 | 0.63 |
| SMBN | 0.20 | 0.55 | -0.09 | 0.60 | -0.06 | 0.12 | 0.44 | 0.40 | 0.32 | -0.32 | 0.63 | 1.00 |

SOC: Soil organic carbon, MO: Moisture, TN: Total nitrogen, TP: Total phosphorus, TK: Total potassium, AN: Available nitrogen, AP: Available phosphorus, AK: Available potassium, Temperature: Soil temperature, SMBC: Soil microbial biomass carbon, SMBN: soil microbial biomass nitrogen.
