# Supplementary material for: Response of soil microbial community structure and function to different altitudes in arid valley in Panzhihua, China
Source: BMC Microbiol. 2022 Apr 2;22:86. doi: 10.1186/s12866-022-02500-6 (PMC8976301; doi:10.1186/s12866-022-02500-6)
Supplement: Supplementary file 3 — Additional file 3: Table S2. The relationships between community composition and standardized soil properties at low (1600 m a.s.l.), medium (1800 m a.s.l.) and high (2000 m a.s.l.) altitudes, tested using distance-based redundancy analysis. [file 12866_2022_2500_MOESM3_ESM.docx]

Table S2. The relationships between community composition and standardized soil properties at low (1600 m a.s.l.), medium (1800 m a.s.l.) and high (2000 m a.s.l.) altitudes, tested using distance-based redundancy analysis.

|  | Low |  | Medium |  | High |  |
| --- | --- | --- | --- | --- | --- | --- |
|  | F | Pr(>F) | F | Pr(>F) | F | Pr(>F) |
| pH | 2.8-3.9 | **0.003-0.021** | 1.1-1.4 | 0.20-0.39 | 0.72-1.1 | 0.39-0.76 |
| SOC | 0.57 | 0.83 | 0.69 | 0.79 | 0.91 | 0.55 |
| MO | 1.4-2.1 | 0.06-0.20 | 0.63-0.81 | 0.60-0.78 | 0.94-1.3 | 0.21-0.48 |
| TN | 1.05 | 0.37 | 1.98 | **0.03** | 1.20 | 0.27 |
| TP | 1.91 | 0.07 | 1.15 | 0.34 | 1.35 | 0.18 |
| TK | 0.61 | 0.83 | 0.45 | 0.95 | 0.74 | 0.74 |
| AN | 1.57 | 0.14 | 1.02 | 0.42 | 0.51 | 0.97 |
| AP | 0.86 | 0.50 | 0.87 | 0.58 | 0.61 | 0.85 |
| AK | 1.98 | 0.09 | 0.93 | 0.53 | 0.64 | 0.86 |
| Temperature | 2.00 | **0.049** | 0.86 | 0.57 | 1.38 | 0.18 |
| SMBC | 0.85 | 0.61 | 1.07 | 0.40 | 1.75 | **0.049** |
| SMBN | 0.98-2.1 | **0.029**-0.45 | 0.56-1.3 | 0.20-0.91 | 0.72-1.7 | 0.09-0.78 |

SOC: Soil organic carbon, MO: Moisture, TN: Total nitrogen, TP: Total phosphorus, TK: Total potassium, AN: Available nitrogen, AP: Available phosphorus, AK: Available potassium, Temperature: Soil temperature, SMBC: Soil microbial biomass carbon, SMBN: soil microbial biomass nitrogen.
